# Microfluidic Chip-Based Induced Phase Separation Extraction as a Fast and Efficient Miniaturized Sample Preparation Method

**DOI:** 10.3390/molecules26010038

**Published:** 2020-12-23

**Authors:** Yao Shen, Bo Chen, Han Zuilhof, Teris A. van Beek

**Affiliations:** 1 Key Laboratory of Chemical Biology and Traditional Chinese Medicine Research, Ministry of Education, Hunan Normal University, Changsha 410081, China; lvy33@163.com (Y.S.); dr-chenpo@vip.sina.com (B.C.); 2 Laboratory of Organic Chemistry, Wageningen University, Stippeneng 4, 6708 WE Wageningen, The Netherlands; han.zuilhof@wur.nl

**Keywords:** fast sample clean-up, IPSE, miniaturization, green analytical chemistry, μLPME, low solvent consumption, microfluidics, on-chip separation, TCM, *Scutellaria baicalensis*

## Abstract

Induced phase separation extraction (IPSE) is an efficient sample clean-up technique that can replace liquid-liquid extraction (LLE). The purpose of this study was to miniaturize IPSE by carrying it out in a microfluidic chip. An IPSE chip was designed and evaluated for its ability to separate and purify samples on a microscale. The 5 × 2 cm chip was fed with a solution of polar to non-polar model compounds in acetonitrile-water (1:1). In the 100 µm wide and 40 µm deep microchannels, the sample solution was efficiently separated into two immiscible phases by adding a hydrophobic solvent as inducer. Analytes present in the sample solution each migrated to their own favorable phase upon phase separation. After optimization, extraction and fractionation were easily and efficiently achieved. The behavior of analytes with a pH-dependent partitioning could be influenced by adjusting the pH of the sample solution. *Scutellaria baicalensis* extract, used in Traditional Chinese Medicine (TCM), was successfully separated in aglycones and glycosides. In this microscale system, the sample and solvent consumption is reduced to microliters, while the time needed for the sample pretreatment is less than one minute. Additionally, the extraction efficiency can reach up to 98.8%, and emulsion formation is avoided.

## 1. Introduction

Most chemical samples are too complex for direct analysis by, for instance, HPLC or GC, and require one or more steps of sample preparation (syn. sample clean-up) [1]. The exact sample prep methodology used varies with the type of sample and type of analyte, e.g., alkaloids [2], fragrances [3] or Traditional Chinese Medicines [4]. In comparison with sampling, the actual final analysis and subsequent data analysis, sample preparation is by far the most time-consuming step. Often manual labor is involved, making it also expensive and error-prone. Majors stated in 2015 that not that much had changed in the preceding 25 years: “Many laboratories still use age-old, time-consuming, manual, labor-intensive sample preparation methods that can still be a source of errors” [5]. Thus, there is much interest in the development of more efficient, less labor-intensive, automated and greener sample preparation methods. One possibility to achieve several of these goals simultaneously is the miniaturization of sample preparation by the application of microfluidic chip-based methods [6,7,8,9,10].

A proper application of microfluidics can generate a lot of advantages in chemical analysis, such as saving solvents, time and labor, and potentially providing a high degree of integration and automation. Some of the traditional sample pretreatment methods have been successfully integrated into microfluidic chips, for example: mixing [11], liquid-liquid extraction (LLE) [12,13,14,15,16], solid phase extraction (SPE) [17,18] and liquid membrane transport extraction [19,20,21]. Thus, it is possible to carry out a variety of chemical processes that are usually done with beakers, flasks and separatory funnels by means of microfluidic chips. Overall lab-on-a-chip techniques ensure that experiments are simpler, more efficient, more environmentally friendly and less harmful for the researcher or analyst. Earlier we reported on two parallel LLE steps in a continuous-flow 3-phase chip [12,22]. On a microscale, the high interfacial surface area between two immiscible liquids is one of the features that make multiphase microfluidics very interesting for analytical chemists. As Reynolds numbers are low, the multiphase flow is laminar, causing molecules to move rapidly from one fluid to the other by diffusion and affinity. This fast mass transfer in microchannels makes chip-based LLE timewise more efficient than macroscale LLE. In a 3-phase chip, due to polarity differences and a pH gradient, basic analytes migrate from a crude aqueous sample solution via an intermediate organic solvent to a much cleaner aqueous acidic acceptor fluid [12,22].

Induced phase separation extraction (IPSE, also known as phase transition extraction (PTE) [23] or solvent-induced phase separation extraction (SIPSE) [24] or solvent-induced phase transition extraction (SIPTE) [25]) is a quick and efficient alternative for LLE. In essence, a water-miscible organic solvent, e.g., acetonitrile, is separated in seconds from water by adding salt [26,27], or a hydrophobic solvent [25], or by cooling the solution to subzero temperatures [28]. Ideally, analytes and matrix components almost instantaneously migrate to their preferred and different phases. There are nice applications of IPSE such as in the analysis of various drugs from plasma [29], the separation of glycosides and aglycones occurring in the medicinal plant *Scutellaria baicalensis* [23], profiling of phytohormones in plants [30], analysis of illegal cationic dyes at low ppb level in foods and surface waters [31] and biomonitoring of organophosphate flame retardants [25].

However, a significant disadvantage of IPSE up till now is that, like traditional LLE, it requires many manual manipulations and is thus difficult to automate. If the IPSE process could be miniaturized to chip-size, automation and later hyphenation with extraction, chromatography or detectors would become feasible, and the solvent consumption could be reduced by three orders of magnitude. Altogether this would create an alternative efficient and environmentally friendly sample pretreatment device at the µL scale. The first fundamental step in this process is to ascertain whether chip-based IPSE is possible at all. Thus, the main aim of this research was downscaling of the IPSE technique using a hydrophobic solvent so that it can be carried out in a microfluidic chip. This encompassed the design and production of the microfluidic chip, basic tests and optimization of solvents and flows using various model compounds, comparison with macroscale IPSE and successful application in the sample preparation of a real-life sample. The results are reported herein.

## 2. Results

### 2.1. Chip Design and Proof of Principle

To carry out miniaturized IPSE using a hydrophobic solvent in a chip, we considered that a design should contain two inlet channels, namely one for the sample solution (in acetonitrile-water) named “Inlet 2” and one for the inducer (hydrophobic solvent) named “Inlet 1”. These two channels should meet and mix for some time to trigger phase separation. Based on our earlier experiences with IPSE [23,29,31], ~10 s should be sufficient to realize initial phase separation and formation of a plug flow. A flow of 1 µL/min in a microchannel of 4 cm length, 100 µm width and 40 µm depth corresponds with a residence time of 12 s. Thus, in the chip design, Inlet 1 and Inlet 2 were placed opposite to each other followed by a channel of 4 cm length. Again, based on our earlier experiences with obtaining stable laminar flows of immiscible liquids in 3-phase chips [12], a 6.4 cm long 2-phase channel separated by small pillars was added to realize full phase separation plus equilibrium of the analytes in the two laminar phases, i.e., complete extraction.

**Figure 1:**
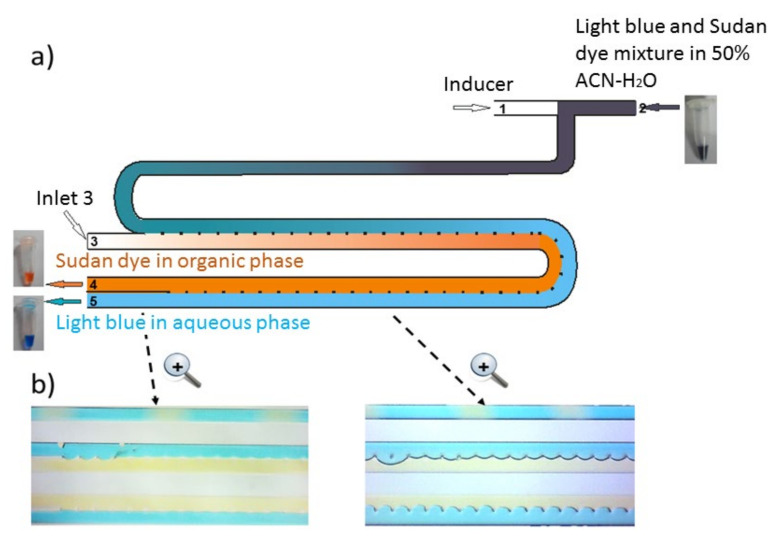
Schematic representation of the induced phase separation extraction chip: (**a**) lay-out of the IPSE chip when filled with a dye mixture (Indigo blue and Sudan red); (**b**) photos of parts of the IPSE chip while separating a blue-orange dye mixture by using the mixed inducer (dichloromethane–butyl acetate (2:8)).

To help converting the initial plug flow at the end of the initial channel of 4 cm, one of the two parallel channels of 6.4 cm was selectively made hydrophobic while the other remained hydrophilic. The combination of surface tension and pillars separates the aqueous and organic phases. At the two outlets of the chip, pure aqueous and organic phases could be collected for further off-line or on-line analysis. Finally, for greater flexibility during method development, it was decided to add an extra inlet channel (Inlet 3) where the single channel that contains the mixed aqueous and organic components splits in the two parallel, pillar-divided channels. This allows for the infusion of an auxiliary organic flow but, if not needed, this inlet can also be blocked. Altogether this led to microfluidic chips made according to the design depicted in Figure 1.

Figure 1a shows schematically how a dye mixture behaves in the IPSE chip. The color of the dye mixture prior to infusion is dark blue, while after separation in the IPSE chip, the organic phase from Outlet 4 is orange (non-polar Sudan dye) and the aqueous phase from Outlet 5 is clear blue (polar Indigo blue dye). In the upper channel of 4 cm length in Figure 1b, the plug flow can be clearly observed while in the lower parallel channels of 6.4 cm length, laminar flows are present. The left photo in Figure 1a zooms in on the outlet part, showing well-separated orange and blue phases each eluting from their own outlet, i.e., proof of principle of a working design was obtained. Next several parameters influencing the IPSE process, including flow rates and inducer-solvent composition, were systematically investigated for their effect on phase separation and extraction efficiency.

### 2.2. Inducer Optimization

#### 2.2.1. Single Solvent Inducer

To bring about phase separation between the less polar, aprotic acetonitrile and the highly polar water, initially a small amount of a single hydrophobic inducer was added, i.e., the non-oxygenated solvents dichloromethane or chloroform, or the oxygenated solvents ethyl acetate or butyl acetate, as these have been successfully applied before [29]. Auxiliary Inlet 3 was kept blocked, and 0.20 µL/min of inducer and 0.80 µL/min of sample were infused. In all cases segmented (plug) flow when infusing a sample of Sudan red (in organic phase) and Indigo blue dye (in aqueous phase) was observed in the mixing channel. This proves phase separation occurred in this channel. Non-oxygenated solvents gave a more pronounced segmented flow, i.e., better initial phase separation, than oxygenated solvents, which is consistent with earlier obtained macroscale data.

However, stable dual laminar flows in the two-phase parallel channels could not be achieved when using chloroform (η = 0.57 cP), dichloromethane (η = 0.44 cP) or ethyl acetate (η = 0.45 cP). The most likely reason for this is the significant viscosity difference between the aqueous and organic phases. Due to this, the aqueous phase (mostly water (η = 1.00 cP) with some acetonitrile (η = 0.37 cP)) will flow slowly, and the organic phase (acetonitrile with chloroform or dichloromethane or ethyl acetate) fast, which finally results in a non-laminar flow in the 2-phase channel part [32]. Thus, using an inducing solvent with a viscosity closer to that of water (η = 1.00 cP), like butyl acetate (η = 0.74 cP) or hexyl acetate (η = 1.07 cP), might offer a solution to this problem. However, butyl and hexyl acetate, were observed to possess a lower inducing efficiency, i.e., less acetonitrile separated from the uniform acetonitrile-water sample solution. Thus, the volume of the organic phase was considerably lower than that of the aqueous phase, which in turn created problems when collecting the two phases at the chip outlets. Ideally the phase ratio should be 1:1 as the two parallel channels have equal dimensions. Therefore, it was decided to revert to the use of non-oxygenated solvents as inducer (increases % organic phase) and at the same time opening Inlet 3 to introduce a more viscous solvent (butyl acetate or hexyl acetate) as an auxiliary fluid.

With the help of the auxiliary fluid infused in Inlet 3, stable parallel laminar flows in the two-phase parallel channels could be realized using non-oxygenated solvents as inducer. Various flow rates for sample, single inducer and auxiliary fluid were investigated: 0.70–1.0 µL/min for sample, 0.05–0.40 µL/min for inducer and 0.10–0.50 µL/min for auxiliary flow. Stable laminar flows in the two parallel channels were obtained when flow rates of 0.80 µL/min, 0.20 µL/min and 0.40 µL/min were used for the sample, inducer and auxiliary fluid, respectively. A drawback of this approach was, however, that relatively much halogenated solvent (0.20 µL/min) was needed and that the organic phase contained relatively little acetonitrile and much auxiliary solvent. This created a large polarity difference between the organic and aqueous phases, which in turn influenced the analyte partitioning. Additionally, it can complicate the injection of the organic phase into a reversed phase LC column in the case of future hyphenation with HPLC.

#### 2.2.2. Mixed Solvent Inducer

**Figure 2:**
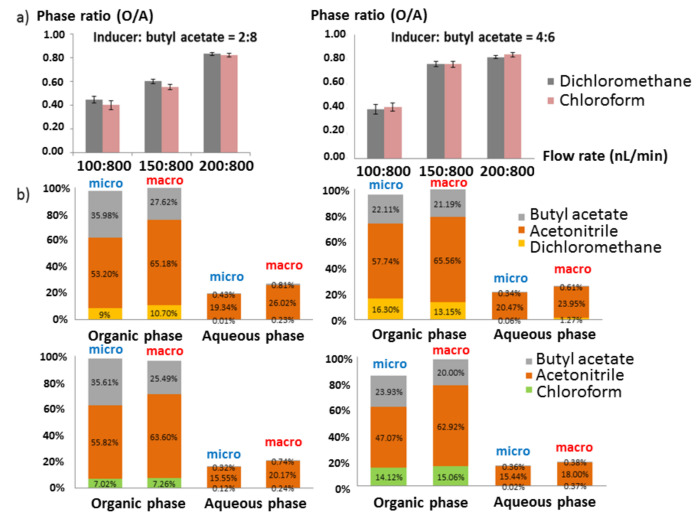
(**a**) Phase ratio as a function of inducer flow rates (0.10, 0.15 and 0.20 µL/min), nature of inducer (dichloromethane or chloroform) and volume ratio of inducer and butyl acetate (2:8 or 4:6); (**b**) relative volume percentages of collected organic and aqueous phases at microscale (IPSE chip) and macroscale (Eppendorf tube) for both dichloromethane (upper) and chloroform (bottom) used at the optimal flow conditions. Volume ratio of inducer and butyl acetate is 2:8 (left) and 4:6 (right).

Thus, mixed inducers consisting of a non-oxygenated solvent such as chloroform or dichloromethane, and an oxygenated solvent such as butyl acetate or hexyl acetate at various flow rates were investigated while Inlet 3 was kept blocked, that is, no auxiliary flow was used. As expected, stable laminar flows in the two-phase parallel channels could be observed because of the simultaneous contribution of a highly efficient phase separation-inducing solvent and a more viscous solvent. Also, the acetonitrile % in the organic phase was high. The observed phase separation and the solvent distribution in each collected phase were used as the criteria to select the best composition and flow rates. Two different ratios (2:8 and 4:6) of non-oxygenated solvent to oxygenated solvent, two different non-oxygenated solvents (dichloromethane and chloroform) and three different ratios of inducer to sample flow rate (0.10:0.80, 0.15:0.80 and 0.20:0.80 µL/min) were tested (see Figure S1a). The ratio of the volumes of the organic and aqueous phases collected is shown in Figure 2a. When the inducer flow rate was increased from 0.10 to 0.20 µL/min while keeping the sample flow constant at 0.80 µL/min, the volume ratio of organic phase to aqueous phase increased from 0.4 to >0.8 for both chloroform and dichloromethane. In addition, when setting the flow rate at 0.20 µL/min for the inducer and 0.80 µL/min for the sample, the most stable laminar flow was obtained.

The total flow rate was not increased further, as higher flow rates would reduce the contact time below 10 s, and this amount of time is needed for a proper mixing of inducer and sample solution, formation of plug flow, complete phase separation and a high extraction yield. The organic and aqueous effluents were collected from each outlet (*n* > 3) and the relative solvent composition (%*v*/*v*) of each effluent was determined by gas chromatography (GC) (Figure 2b). Based on the flow rates, solvent ratios and collected volumes of aqueous and organic phases, and under the assumption that almost all of the water will end up in the aqueous phase and almost all of the inducer and butyl acetate end up in the organic phase, one can calculate that the ~450 µL of organic phase will consist of ~250 µL of acetonitrile, ~160 µL of butyl acetate and ~40 µL of dichloromethane, and the ~550 µL of aqueous phase of ~150 µL acetonitrile and 400 µL of water.

The data presented in Figure 2b correspond well with this prediction. Acetonitrile is the major solvent in the organic phase, which proves that phase separation occurred in the chip. When the proportion of non-oxygenated solvent in the inducer was increased from 20% to 40% (Figure 2a, right), there was neither a big increase in the separation of the acetonitrile from the water (see Figure S1b) nor a big change in phase ratio, but twice as much halogenated solvent was used. As a lower consumption of halogenated solvents is preferred from an environmental point of view, and also considering a more stable laminar flow in the two parallel channels, 20% chloroform or dichloromethane was chosen as the optimal composition for the mixed inducer. The best condition for using the mixed inducer in the IPSE chip, therefore, is 20% of dichloromethane in butyl acetate as inducer at a flow rate of 0.20 µL/min in combination with a sample flow rate of 0.80 µL/min of acetonitrile—water (1:1).

Comparing the IPSE chip data to our earlier macroscale IPSE data without butyl acetate [29], the phase separation efficiency in the IPSE chip appears to be similar (Figure 2b). There was still some water (around 5%) remaining in the organic phase and around 20% of acetonitrile was still present in the aqueous phase (Figure 2b). For an even better comparison with the chip-based experiments, IPSE of 50% acetonitrile-water with the same mixed inducer was also carried out at a macroscale (Eppendorf tube) and the relative solvent distribution in each phase was analyzed by the same GC method. Results (Figure 2b) show that both micro and macroscale gave similar relative solvent distributions, although the percentage of acetonitrile was a bit lower and the percentage of butyl acetate a bit higher in the microscale IPSE. This could be due to non-equilibrium effects (kinetics of phase separation) at the microscale or occasionally by the exiting of a droplet from the wrong channel.

### 2.3. Extraction Efficiency

#### 2.3.1. Model Compounds

**Figure 3:**
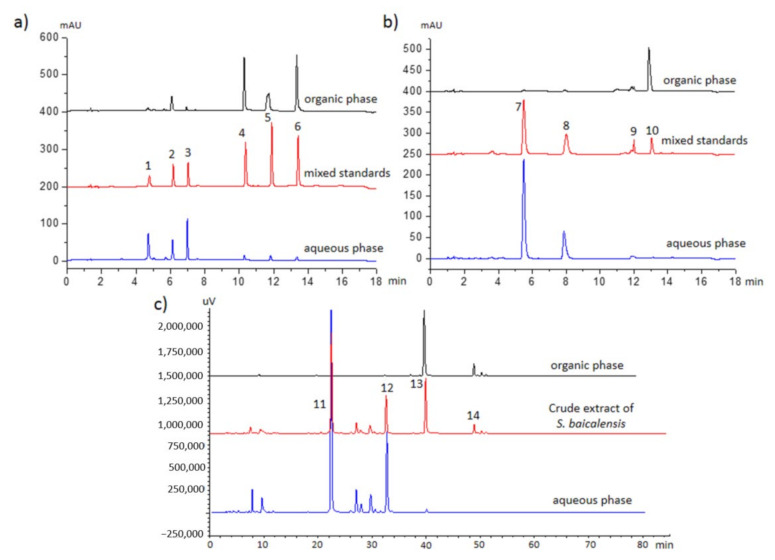
(**a**) HPLC profiles of the mixture of six model compounds (chlorogenic acid (peak 1), epigallocatechin gallate (peak 2), rutin (peak 3), quercetin (peak 4), santonin (peak 5) and alizarin (peak 6)), and the corresponding organic and aqueous phases collected at the outlets of the IPSE chip; (**b**) HPLC profiles of the mixture of four acidic or basic model compounds (4-hydroxybenzoic acid (peak 7), syringic acid (peak 8), emetine (peak 9), and vincamine (peak 10)) and the corresponding organic and aqueous phases collected at the outlets of the IPSE chip; (**c**) HPLC profiles of the mixture of *S. baicalensis* extract and the corresponding organic and aqueous phases collected at the outlets of the IPSE chip; baicalin (peak 11), wogonoside (peak 12), baicalein (peak 13), wogonin (peak 14). For structures, see Figure 4.

The separation efficiency of the IPSE chip was characterized under the optimized conditions. Theoretically 100% separation efficiency is never attainable with any LLE-based system, nor will it be possible to get a good separation, like >90% in either phase for all analytes in a complex sample, with a single solvent system. However, based on our earlier experience [29], usable and fast results can be obtained reproducibly by IPSE. For the characterization, a set of six model compounds of natural origin of different polarities was used: chlorogenic acid, rutin, epigallocatechin gallate, quercetin, santonin and alizarin (see Figure S2 for structures, numbers correlate with peak numbers in Figure 3a,b). Their respective calculated log D values at pH = 7 are −3.04, −1.92, 0.57, 1.08, 1.15 and 2.05 [33]. Figure 3a shows the chromatograms of a mixture of the six model compounds and the collected organic and aqueous phase exiting the IPSE chip. The two most polar compounds, chlorogenic acid and rutin, were mostly found in the aqueous phase, while the three non-polar compounds, quercetin, santonin and alizarin, were almost exclusively present in the organic phase. The log D value of epigallocatechin gallate falls in between those of quercetin and rutin, and thus it turns up in both phases. The extraction efficiency for each compound both in the IPSE microfluidic chip and by IPSE in an Eppendorf tube is shown in Table 1.

With the exception of chlorogenic acid and santonin, the extraction efficiency on micro and macroscale is comparable. The reproducibility of IPSE, expressed as relative standard deviation (RSD) is ~3.5% and ~2% for chip and macroscale, respectively. These differences in extraction efficiency and RSD are most likely related to the fact that the IPSE process in the chip is much faster (<30 s). Once the two phases exit the chip, there is no more opportunity to reach equilibrium. Additionally, for acidic compounds like chlorogenic acid log D varies a lot between pH 3 to 8 [33] – small local changes in pH will influence its partitioning. What is furthermore interesting to observe, is that the partitioning behavior of analytes in the IPSE process does not correspond with the partitioning behavior of those same analytes in RP-HPLC. Epigallocatechin gallate elutes before rutin (Figure 3a) on an RP-HPLC column, yet rutin is for 94% present in the aqueous phase and epigallocatechin gallate only for 57%. This different selectivity offers interesting possibilities for IPSE to separate analytes during sample pretreatment, which co-elute on an RP-column.

#### 2.3.2. pH Effect on Acidic and Basic Compounds

For amines, acids and phenols, a change in the sample pH will greatly affect their extraction behavior and in turn this could create valuable separation opportunities. To test this, the IPSE behavior of two acids and two amines: syringic acid, 4-hydroxybenzoic acid, vincamine and emetine (see Figure S2 for their structures) was studied. At pH 10, the two acids were mostly expected in the aqueous phase and the two alkaloids mostly in the organic phase. All four compounds were injected at pH 3 and 10, and both phases were collected and off-line investigated by HPLC (Figure 3b). Both acids and vincamine behaved as expected (Table 2), but emetine showed erratic behavior at both pHs and at both micro and macroscale. Overall the extraction efficiencies at micro and macroscale were comparable. High extraction efficiencies of 94–99% into one phase were observed for syringic acid, 4-hydroxybenzoic acid and vincamine at pH 9.95. During the extraction of alkaloids by either traditional LLE and by macroscale IPSE, persistent emulsions are frequently formed.

In contrast, during the extraction of alkaloids by the IPSE chip, clean and stable laminar flows were observed at both pHs without emulsion formation. This is a significant advantage of IPSE in a chip versus traditional IPSE in a tube. This advantage would enable a reliable application of an IPSE chip in automated on-line sample clean-up.

#### 2.3.3. Efficiency Comparison of Microfluidic IPSE and Microfluidic LLE

The obtained extraction efficiencies of the model compounds, which are predominantly, i.e., 90–100%, found in one phase (see Section 2.3.1 and Section 2.3.2) varied from 89.6% (chlorogenic acid) to 98.8% (4-hydroxybenzoic acid) with an average recovery of 94.5%. This compares favorably with LLE efficiencies obtained in microfluidic chips. Some results from literature are 40% and 92% for tanshinone after 20 and 80 s residence time respectively (3-phase chip) [34], 79.5% for strychnine (3-phase chip, 25 s) [12], 92% for strychnine (3-phase chip, 25 s) [22], 63.4% for sanguinarine (2-phase chip) [35], 20% for sanguinarine (3-phase chip) [15] and 43.6% for paclitaxel (3-phase chip) [16]. Thus, on-chip IPSE appears at least competitive with on-chip LLE: recovery is similar or better, required time is approximately the same and operation of the 2-phase IPSE chip is more robust than that of a 3-phase chip.

### 2.4. Real-Life Sample Application

Finally, the optimized chip was tested with an extract of the plant *Scutellaria baicalensis*. This species is an important constituent of Traditional Chinese Medicines (TCMs) and has earlier been analyzed by means of macroscale IPSE [23]. Figure 3c shows the chromatograms of the separation of aglycones and glycosides present in *S. baicalensis* by the IPSE microfluidic chip. Aglycones, like baicalein (**13**) and wogonin (**14**), were almost exclusively found in the organic phase, while glycosides such as baicalin (**11**) and wogonoside (**12**) were almost exclusively present in the aqueous phase. This outcome is comparable with the macroscale result [23]. This application proves that the IPSE chip works well for more complex matrixes. Furthermore, it is again noteworthy that the elution behavior in IPSE can deviate from RP-HPLC elution behavior. Wogonoside (**12**) and baicalein (**13**) are in different IPSE fractions in spite of the fact that their retention times are relatively close (Figure 3c). This selectivity could improve the analysis of complex natural mixtures such as TCMs.

## 3. Discussion

An induced phase separation extraction (IPSE) microfluidic chip was newly designed and tested. It can be used for efficient miniaturized sample pretreatment much like microfluidic LLE. The developed IPSE chip could successfully separate acetonitrile—water (1:1) sample solutions into individual organic and aqueous phases by adding 20% hydrophobic inducer. The behavior of analytes in the sample solution is correlated to their log D values, but less so to the elution order in RP-HPLC. This different selectivity makes the combination IPSE-HPLC interesting for compounds, which co-elute in RP-HPLC. Adapting the pH for analytes with a pK_a_ value between 4 and 10 can further expand this difference in selectivity. The whole process can be finished in 30 s at low flow rates: 0.2 µL/min for inducer (20% of dichloromethane in butyl acetate), and 0.8 µL/min for sample solutions. The extraction efficiency of the IPSE chip is equal or even higher than that of macroscale IPSE carried out in Eppendorf tubes as shown by the analysis of model compounds. The average extraction efficiency of 94.5% of the developed IPSE chip compares favorably with the reported efficiencies of LLE microfluidic chips [12,15,16,22,34,35].

The model compounds of variable polarity showed that—similar to LLE—IPSE can never achieve a quantitative partitioning of *all* analytes in one particular fraction, epigallocatechin gallate and emetine being examples. Although not investigated in this chip development study, we expect that—again similar to LLE—the partitioning of an individual analyte can be changed by adapting the flow rates and relative percentages of water, acetonitrile and inducer. Possibly acetonitrile could be exchanged by another moderately polar (partially) water-miscible solvent like acetone, *tert*-butanol, tetrahydrofuran, methyl acetate or mixtures thereof. The reproducibility (RSD ≈ 3.5%) of chip-based IPSE separations is similar to that of manual separatory funnel-based LLE separations, and allows applications in quantitative analyses. As a real-life sample, an extract of *S. baicalensis* was well separated by the IPSE chip into its respective aglycones and glycosides, and the results were virtually identical with those obtained by macroscale IPSE [23]. Obtaining positive results with a real sample (Figure 3) is considered to be an essential development step for microfluidic devices aiming at sample clean-up [6]. When comparing our earlier 3-phase sample pretreatment chip [12] with the IPSE chip, the 2-phase IPSE chip appears more robust.

Future research we envisage for the IPSE chip are hyphenation to a miniaturized extraction cell (upstream), HPLC or MS (both downstream) for automated on-line analysis of, e.g., complex plant samples to save time and increasing reproducibility while at the same time avoiding the introduction of impurities and possible analyte degradation. The exit flow of 0.5 µL/min of one channel is ideal for combining chip-based IPSE with on-line microLC (full 100 nL loop) or UHPLC (partial 5 µL loop).

## 4. Materials and Methods

### 4.1. Materials and Instruments

Chlorogenic acid, epigallocatechin gallate, rutin, quercetin (≥95%), santonin (≥99%), alizarin (97%), 4-hydroxybenzoic acid, syringic acid, vincamine and emetine were purchased from Sigma (Zwijndrecht, The Netherlands). Acetonitrile, dichloromethane and chloroform (HPLC grade) were purchased from Biosolve (Valkenswaard, The Netherlands). Hexyl acetate, butyl acetate, ethyl acetate, benzene, chloroform, dichloromethane and formic acid were purchased from Merck (Amsterdam, The Netherlands). Sudan red and Indigo blue were bought from Aladdin (Shanghai, China). Plant material (*Scutellaria baicalensis* Georgi) was purchased from a local pharmacy in Changsha (China).

The IPSE chips were manufactured from borosilicate glass by means of photolithographic fabrication methods (Micronit, Enschede, The Netherlands) based on our own design. The IPSE chip was mounted in a PEEK holder from Micronit and connected to the syringe pumps via Teflon tubing (100 µm, i.d.). Gas tight syringes (500 μL, 3.26 mm i.d.), ferrules, nuts, Luer lock adapters, and filters were purchased from Alltech (Breda, The Netherlands). Syringe pumps (Harvard 11 PicoPlus, dual syringe) were purchased from VWR International (Amsterdam, The Netherlands). Phase separation in the microchip was monitored by means of a DNT digital microscope (Conrad, Oldenzaal, The Netherlands).

The UHPLC was an 1290 Infinity system (Amstelveen, The Netherlands) equipped with binary pumps, autosampler, thermostatted column compartment (25 °C), DAD and column (Agilent Zorbax Eclipse Plus C18, 2.1 × 100 mm 1.8 µm). Mobile phase A: 0.1% formic acid in water; mobile phase B: acetonitrile. Flow rate: 0.20 mL/min. Gradient: 0–2 min 10% B; 2–15 min B increased from 10% to 60%; 15–16 min 60% B; 16.01–18 min 10% B. Wavelength: 254 nm. Injection volume: 1.0 µL. For acidic and basic compounds (polarity is dependent on pH): mobile phase A: 0.1% formic acid in water; mobile phase B: acetonitrile. Flow rate: 0.20 mL/min. Gradient: 0–10 min 10% B; 10.01–15 min 25% B; 15.01–18 min 10% B. Wavelength: 270 nm. Injection volume: 1.0 µL. For *Scutellaria baicalensis* analyses: SPD-M20A (Shimadzu Benelux, ‘s Hertogenbosch, The Netherlands), DAD, and column (Shim-pack VP-ODS 4.6 × 250 mm, 5 μm). Mobile phase A: 0.1% acetic acid in water; mobile phase B: acetonitrile. Flow rate: 1.0 mL/ min. Gradient: 0–5 min 20% B, 5–30 min 20–30% B, 30–60 min 30–60% B, 60–80 min 20% B. Wavelength: 254 nm. Injection volume: 20 µL.

Concentrations of the first set of model compounds in acetonitrile-water (1:1) solution were 49.8, 84.6, 62.9, 32.9, 38.0, and 24.1 µg/mL for chlorogenic acid, epigallocatechin gallate, rutin, quercetin, santonin, and alizarin, respectively. Concentrations of the second set of model compounds in acetonitrile-water (1:1) solution were 55.0, 62.0, 59.0, and 55.0 µg/mL for syringic acid, 4-hydroxybenzoic acid, emetine and vincamine, respectively.

Solvent distribution analysis was carried out by gas chromatography (GC) on a GC 2010 (Shimadzu). Column: DB-1701P, 30 m × 0.25 mm × 0.25 µm, Agilent; temperature program: 0–5 min, 40–100 °C, 5–10 min 100–200 °C; FID: 280 °C. Split ratio: 1:40. Injection volume: 0.1 µL, injection temperature: 180 °C. Relative solvent percentage (*v*/*v*) was calculated by separate calibration curves for each solvent.

### 4.2. IPSE Chip and Chip Modification

An all-glass two-phase chip was used for microscale IPSE. The design is depicted in Figure 1a and further discussed in the Results and Discussion section. To facilitate full phase separation, the two parallel channels of 100 μm width and 40 μm depth are in open connection with one another. In between the parallel channels are pillar structures (semi-toroidal shape, represented by dots in Figure 1a located at equal distances of 120 µm; width at half height is 20 µm), which help to maintain a stable interface. The pillars cover 21% of the potential interfacial contact area thus leaving effectively 79% of the area for direct liquid-liquid contact, i.e., available for extraction. Prior to use, the IPSE chip was first washed with a basic 0.1 M NaOH solution, followed by water, 0.1 M sulfuric acid and again water. After washing, one of the parallel channels (the future organic phase channel) was selectively modified to make it hydrophobic by infusing octadecyltrichlorosilane in hexadecane into one channel and pure hexadecane in the other channel.

### 4.3. Parameter Studies

Regarding the inducer-solvent composition, both single and mixed solvents were investigated by collecting the organic and aqueous phases at exits 4 and 5, followed by off-line GC analysis to study the relative solvent composition (%*v*/*v*). Physical parameters for each used solvent, such as viscosity and polarity were comprehensively considered with the aim of achieving a stable laminar flow in chip. Experiments were carried out in triplicate.

Acetonitrile-water (1:1) solution [29] was used as sample solution during all experiments, and contained initially a non-polar and a polar dye (Sudan red and Indigo blue, respectively) to make it easier to observe the phase separation and extraction during the optimization experiments.

The extraction efficiency was determined by off-line UHPLC analysis in triplicate. The organic and aqueous phases eluting from the two outlets of the IPSE chip, were collected separately. The collected liquids were analyzed every hour (around 30 µL for each). pH buffer: NaHCO_3_-Na_2_CO_3_ buffer 10 mM pH 9.95; acetate buffer 10 mM, pH 3.01. Preparation of real-life sample: 0.50 g powder of *S. baicalensis* was mixed with 50 mL of acetonitrile-water (1:1) solution and sonicated during 40 min followed by filtration through a 0.45 µm membrane.

## Figures and Tables

**Figure 4:**
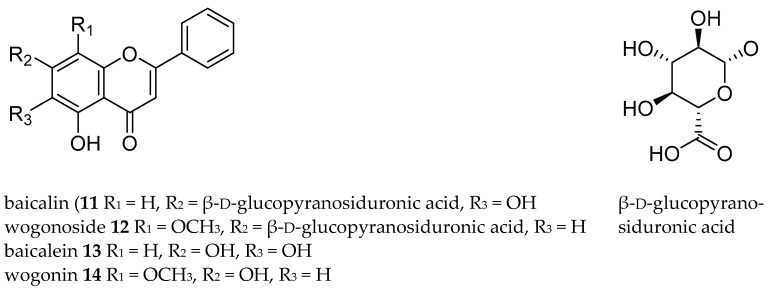
Structures of baicalin (**11**), wogonoside (**12**), baicalein (**13**) and wogonin (**14**).

**Table 1:** Extraction efficiency of six model compounds by IPSE carried out in a microfluidic chip and in Eppendorf tube.

Compounds	Extraction Efficiency of IPSE Chip in % (SD, *n* = 3)		Extraction Efficiency of IPSE in Eppendorf Tube in % (SD, *n* = 3)	
	Organic Phase	Aqueous Phase	Organic Phase	Aqueous Phase
Chlorogenic acid	10.4 (0.89)	89.6 (1.3)	19.1 (0.62)	80.9 (0.64)
Epigallocatechin gallate	43.2 (0.69)	56.8 (1.0)	45.5 (2.5)	54.5 (2.7)
Rutin	6.10 (0.21)	93.9 (1.5)	6.60 (0.039)	93.4 (0.46)
Quercetin	94.0 (2.2)	6.00 (0.37)	94.4 (0.094)	5.60 (0.16)
Santonin	90.7 (1.9)	9.30 (0.37)	93.9 (0.46)	6.10 (0.11)
Alizarin	96.2 (1.9)	3.80 (0.29)	96.8 (0.38)	3.20 (0.10)

**Table 2:** Extraction efficiency of four pH-dependent model compounds by IPSE on micro and macroscale.

Compounds	Extraction Efficiency at pH 3.07 in % (SD, *n* = 3)				Extraction Efficiency at pH 9.95 in % (SD, *n* = 3)			
	IPSE Chip		Eppendorf Tube		IPSE Chip		Eppendorf Tube	
	Organic Phase	Aqueous Phase	Organic Phase	Aqueous Phase	Organic Phase	Aqueous Phase	Organic Phase	Aqueous Phase
4-Hydroxy-benzoic acid	78.7 (9.5)	21.3 (0.76)	72.7 (0.014)	27.3 (0.46)	1.20 (0.085)	98.8 (0.82)	2.20 (0.53)	97.8 (0.91)
Syringic acid	73.5 (9.2)	26.5 (0.64)	67.4 (2.6)	32.6 (0.35)	1.40 (0.056)	98.6 (3.1)	3.00 (0.72)	97.0 (2.5)
Emetine	47.2 (1.8)	52.8 (4.4)	33.6 (0.93)	66.4 (23)	48.5 (10)	51.6 (3.9)	43.1 (4.5)	56.9 (2.4)
Vincamine	19.4 (0.66)	80.6 (2.0)	16.7 (0.18)	83.4 (0.41)	94.00 (7.8)	5.96 (1.8)	97.2 (1.2)	2.81 (0.66)

## Supplementary Materials

The following are available online. Figure S1a: Relative volume percentages of collected organic phases at microscale (IPSE chip) for both dichloromethane (upper) and chloroform (bottom) used at the 3 flow rates; Figure S1b: Relative volume percentages of collected aqueous phases at microscale (IPSE chip) for both dichloromethane (upper) and chloroform (bottom) used at the 3 flow rates; Figure S2: Structures of 10 model compounds used for IPSE efficiency test.

## Sample Availability

Samples of the compounds are not available from the authors.
