# Supplementary material for: Microfluidic Chip-Based Induced Phase Separation Extraction as a Fast and Efficient Miniaturized Sample Preparation Method
Source: Molecules. 2020 Dec 23;26(1):38. doi: 10.3390/molecules26010038 (PMC7796191; doi:10.3390/molecules26010038)
Supplement: Supplementary file 1 [file molecules-26-00038-s001.pdf]

Supplementary Materials belonging to the paper entitled "Microfluidic chip-based induced phase separation extraction as a fast and efficient miniaturized sample preparation method" by Yao Shen, Bo Chen, Han Zuilhof and Teris A. van Beek

Figure S1a. Relative volume percentages of collected organic phases at microscale (IPSE chip) for both dichloromethane (upper) and chloroform (bottom) used at the 3 flow rates.

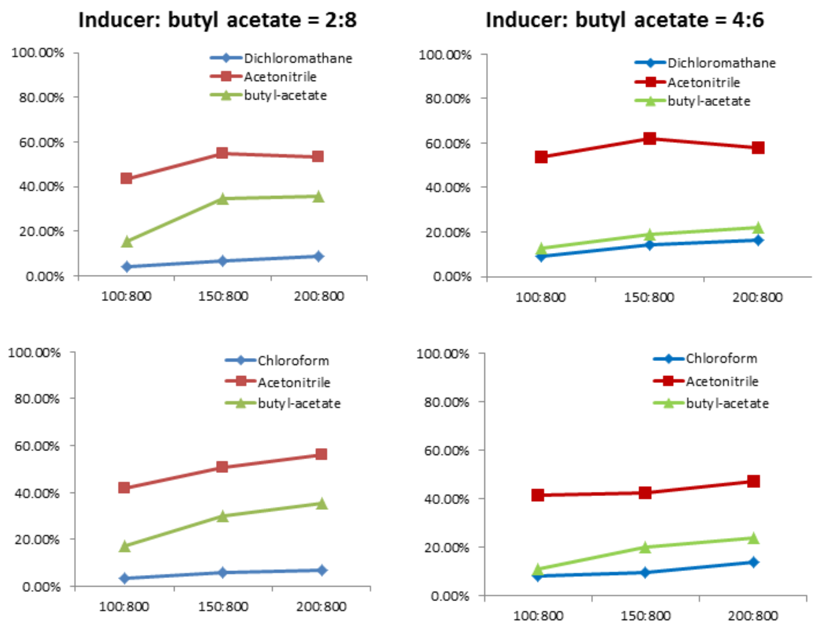

Figure S1b: Relative volume percentages of collected aqueous phases at microscale (IPSE chip) for both dichloromethane (upper) and chloroform (bottom) used at the 3 flow rates.

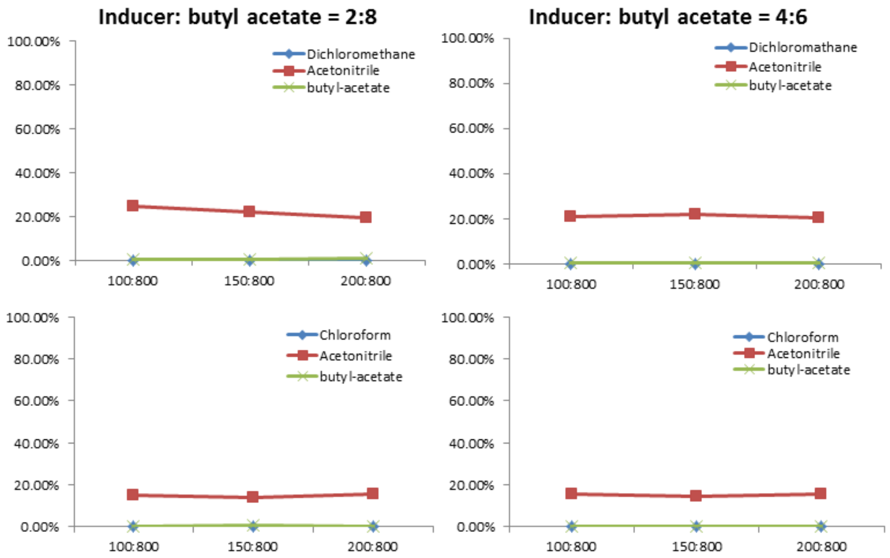

Figure S2: Structures of 10 model compounds used for IPSE efficiency test

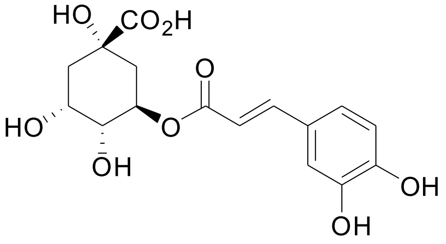

chlorogenic acid

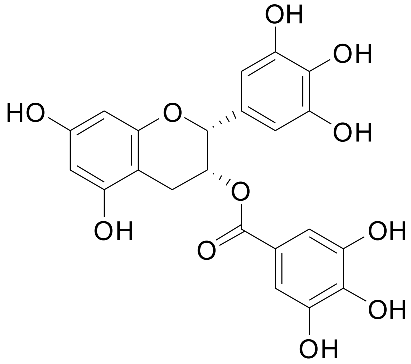

epigallocatechin gallate

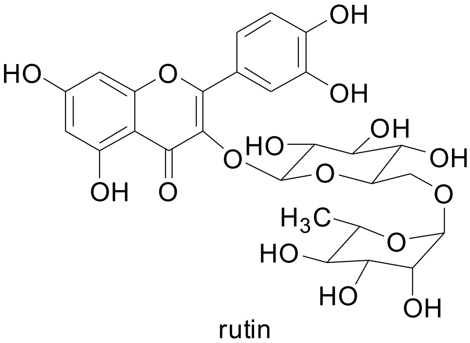

rutin

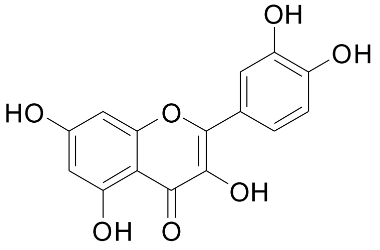

quercetin

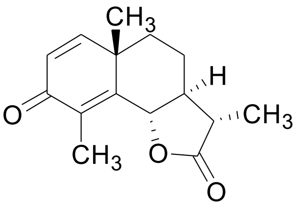

santonin

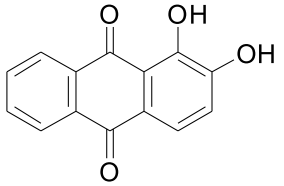

alizarin

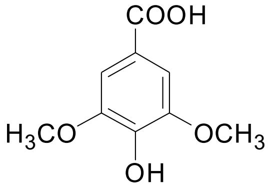

syringic acid

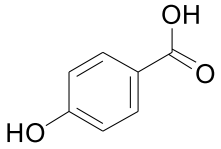

4-hydroxybenzoic acid

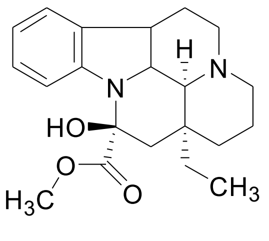

vincamine

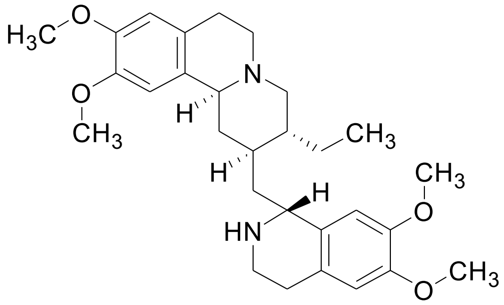

emetine
